# Simultaneous increase in strength and ductility by decreasing interface energy between Zn and Al phases in cast Al-Zn-Cu alloy

**DOI:** 10.1038/s41598-017-12286-7

**Published:** 2017-09-22

**Authors:** Seung Zeon Han, Eun-Ae Choi, Hyun Woong Park, Sung Hwan Lim, Jehyun Lee, Jee Hyuk Ahn, Nong-Moon Hwang, Kwangho Kim

**Affiliations:** 1 Structural Materials Division, Korea Institute of Materials Science, Changwon, 642-831 Korea; 2 Department of Advanced Materials Science and Engineering, Kangwon National University, Chuncheon, 200-701 Korea; 3 Department of Materials Science and Engineering, Changwon National University, Changwon, 641-773 Korea; 4 Department of Materials Science and Engineering, Seoul National University, Seoul, 151-744 Korea; 5 School of Materials Science and Engineering, Pusan National University, Busan, 609-735 Korea

## Abstract

Cast-Al alloys that include a high amount of the second element in their matrix have comparatively high strength but low ductility because of the high volume fraction of strengthening phases or undesirable inclusions. Al–Zn alloys that have more than 30 wt% Zn have a tensile strength below 300 MPa, with elongation under 5% in the as-cast state. However, we found that after substitution of 2% Zn by Cu, the tensile strength of as-cast Al–Zn–Cu alloys was 25% higher and ductility was four times higher than for the corresponding Al–35% Zn alloy. Additionally, for the Al–43% Zn alloy with 2% Cu after 1 h solution treatment at 400 °C and water quenching, the tensile strength unexpectedly reached values close to 600 MPa. For the Al–33% Zn alloy with 2% Cu, the tensile strength was 500 MPa with 8% ductility. The unusual trends of the mechanical properties of Al–Zn alloys with Cu addition observed during processing from casting to the subsequent solution treatment were attributed to the precipitation of Zn in the Al matrix. The interface energy between the Zn particles and the Al matrix decreased when using a solution of Cu in Zn.

## Introduction

Generally, structural metals including Fe-, Cu- and Al-base alloys require high strength to sustain the desired shape and high ductility for easy forming. Additionally, a good combination of strength and ductility in structural materials reflects their toughness for high reliability. The Al-base alloys with their inherent lower density than other metallic alloys would be the best candidates as high specific strength structural alloys^1–5^.

Metallic alloys including Al alloys are usually classified into cast and wrought alloys^1–3,6,7^. The cast alloys are directly formed into the desired shape by casting and appropriate machining without any plastic deformation. High amounts of elements addition in the matrix induce not only strengthening phases, but also inclusions. During solidification, intermetallic compounds are formed that affect the mechanical properties, inevitably leading to a decrease in ductility. Wrought alloys that have a comparatively lower content of alloying elements than cast alloys and thermomechanical treatments, including aging and plastic deformation, may be used to make wires, rods, strips or pipes. In other words, it may be possible to increase the strength and its trade-off property, ductility, simultaneously by thermomechanical processing; e.g., by introducing an alloying element with ultrafine grain^8–12^, nanotwin^13–16^ or marble^17^ structures, or bimodal intermetallic compounds^18^ in the metal matrix.

However, the various abovementioned metallurgical methodologies cannot be implemented because of the addition of high amounts of the second element, which produces low ductility in the cast alloys and limits thermomechanical treatments. Therefore, overcoming the trade-off relationship between strength and ductility is known to be difficult^19^.

**Figure 1:**
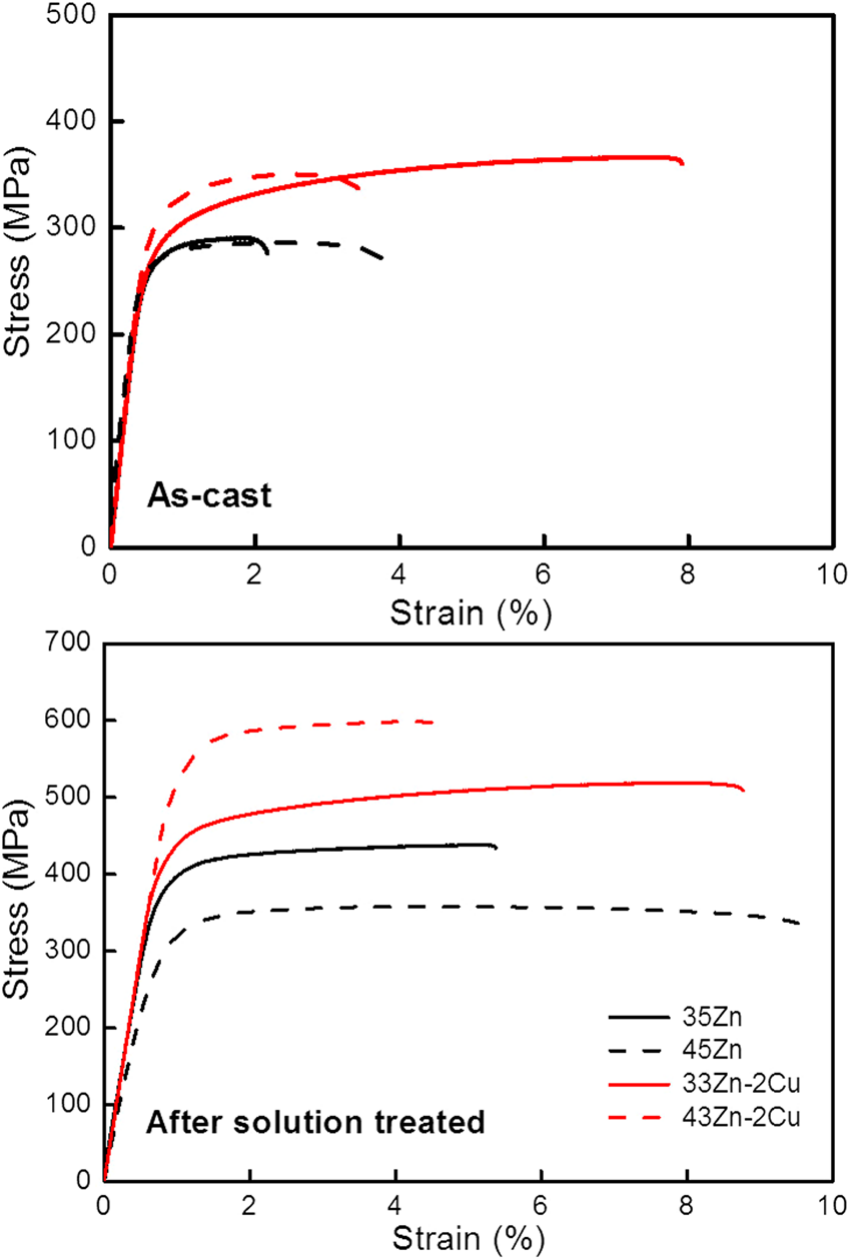
Stress–strain curve of (**a**) as-cast and (**b**) solution-treated Al–Zn alloys without or with 2 wt% Cu. The Al–33Zn alloy with Cu after casting or solution treatment shows increased strength and ductility simultaneously in comparison with the Al–35Zn alloy without Cu. The strength of the Al–45Zn alloy with Cu addition after solution heat treatment is 1.8 times higher than the corresponding Al–45Zn alloy, which contrasts with the notion that solution heating decreases the strength in Al alloys.

We conducted studies on the behaviour of Al–Zn–Cu alloys after casting and solutionizing, and found that their combination of strength and ductility was better than in conventional cast Al alloys (Fig. 1a). Interestingly, the alloy strength increased after solution treatment in contrast to the general expectation that strength should inevitably decrease and ductility should increase in an alloy solution (Fig. 1b). After solution treatment at 400 °C for 60 min. and water quenching of an Al–33Zn–2Cu alloy, we obtained a high-strength and high-ductility combination (515 MPa and 8%, respectively) compared with 435 MPa and 4.5% for the corresponding alloy without Cu. Moreover, the alloy containing Cu had an outstanding combination of strength and ductility after casting (365 MPa and 7%, respectively), which was higher than other alloys in this study. The Cu addition to Al–Zn alloys definitely increased strength or simultaneously improved both strength and ductility at certain compositions during casting and solution treatment (Fig. 1). This unusual behaviour of the Al–Zn alloy with Cu is important for cast Al alloy fabrication because it means that an economical production process consisting only of casting and heat treatment could easily achieve good mechanical properties. Moreover, Zn and Cu are generally known as cheap alloying elements. Based on the relationship between microstructure and mechanical properties, we used density functional theory (DFT) calculations in this study to clarify why adding Cu to an Al–Zn alloy changed the precipitation structure and simultaneously increased strength and ductility during the solidification stage after casting and subsequent solution treatment.

## Results

### Microstructure of Zn precipitates in the Al matrix

**Figure 2:**
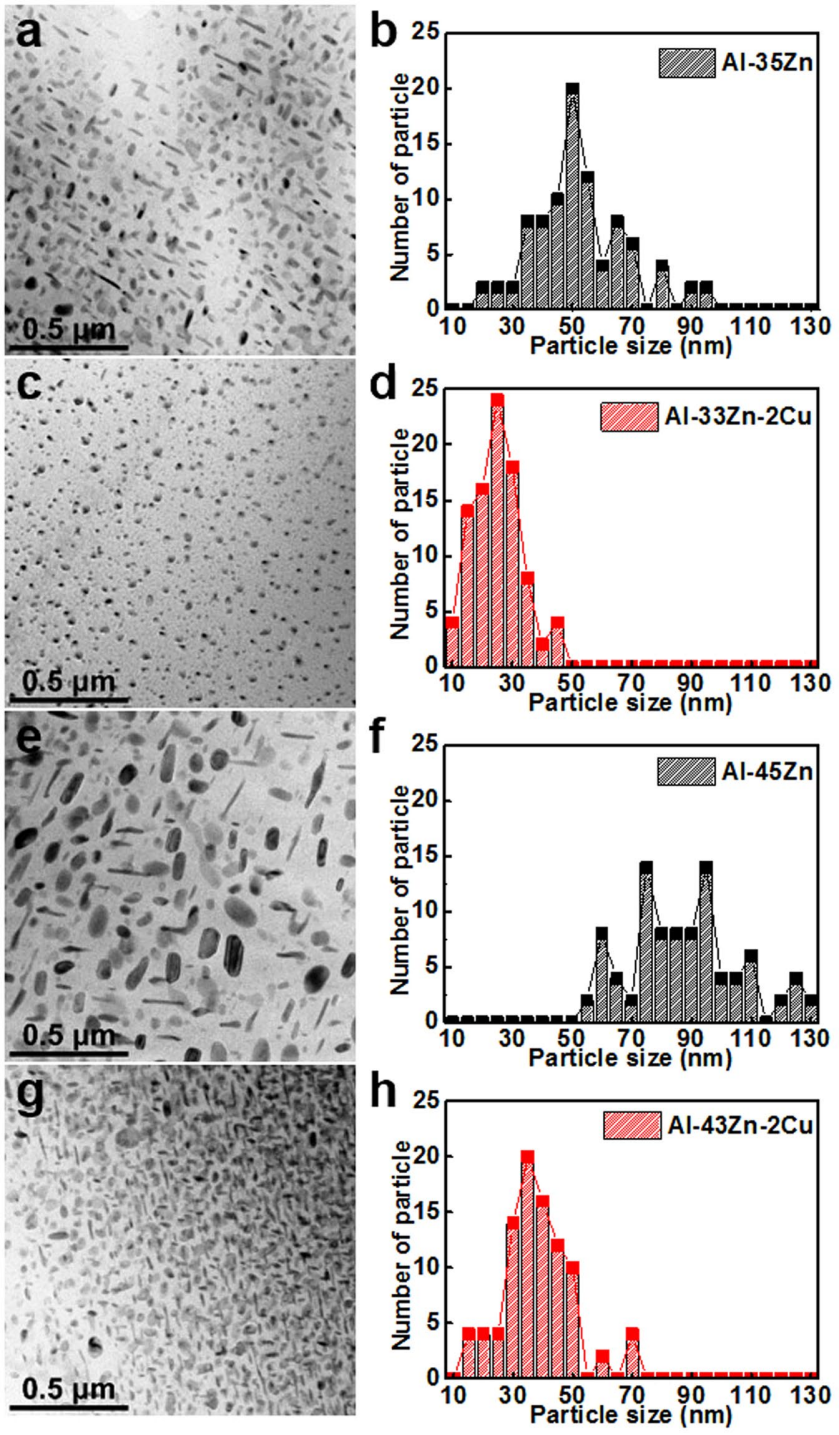
TEM microstructure and particle size distribution of as-cast Al–Zn alloys: (**a**), (**b**) Al–35Zn, (**c**), (**d**) Al–33Zn–2Cu, (**e**), (**f**) Al–45Zn and (**g**), (**h**) Al–43Zn–2Cu. The high Zn content in the Al alloy leads to precipitation during solidification because of the high driving force of precipitation, and the Cu addition to the Al–Zn alloy reduces the size of the precipitates after casting.

**Figure 3:**
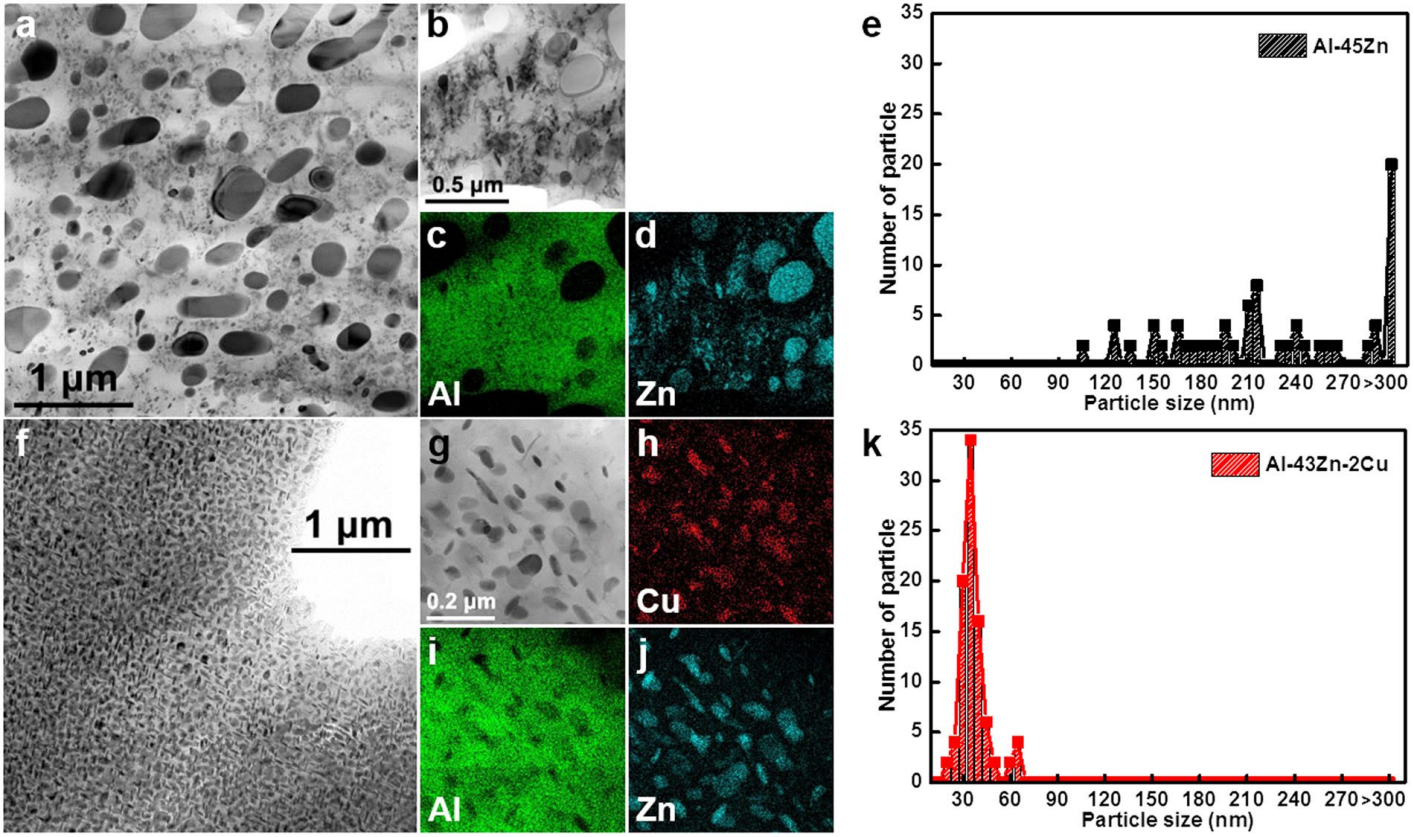
The TEM images and EDS results of solutions treated at 400 °C for 1 h and then water quenched (**a**), (**b**–**d**) Al–45Zn and (**f)**, (**g**–**j**) Al–43Zn–2Cu alloy, and the particle distributions of (**e**) Al–45Zn and (**k**) Al–43Zn–2Cu. The high content of Zn in the Al matrix brought the unstable supersaturated solution and precipitation occurred even under fast cooling.

**Figure 4:**
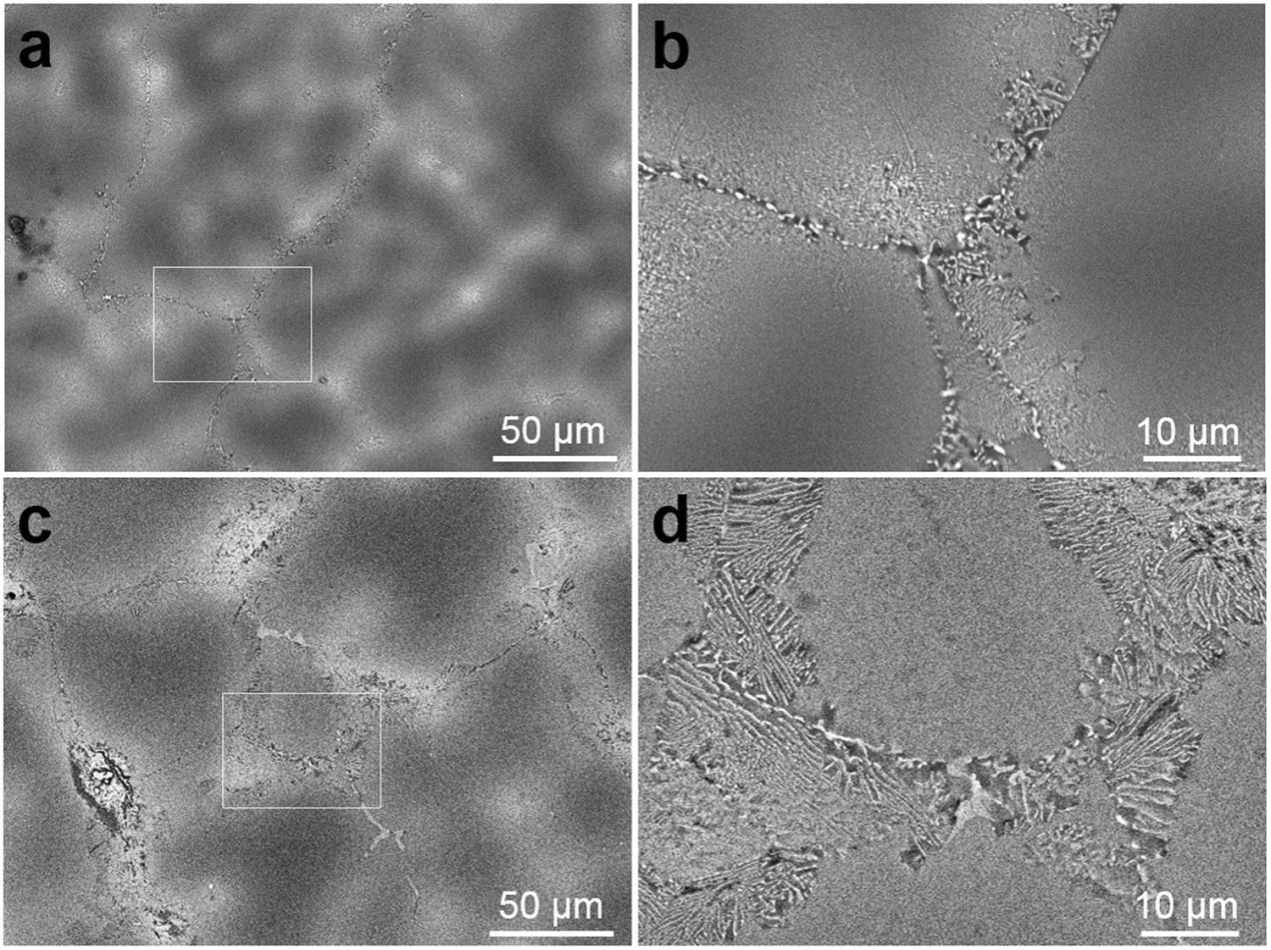
SEM images of as-cast (**a**), (**b**) Al–35Zn and (**c**), (**d**) Al–33Zn–2Cu alloys. The Cu addition to Al–Zn alloy changed the aspect of heterogeneous nucleation at the GBs. Isolated large heterogeneous particles were suppressed and it changed to discontinuous precipitation by Cu addition. The large particles that are believed to be the source of crack initiation were effectively suppressed and the ductility of the Al–33Zn–2Cu alloy increased.

Transmission electron microscope (TEM) images of the microstructure of as-cast Al–Zn alloys with and without Cu are shown in Fig. 2. The microstructure of the Al–Zn alloy with Cu addition showed a reduced average particle size and a uniform distribution after casting. Figure 3 shows the microstructures of Zn particles of Al–45Zn and Al–43Zn alloy with 2 wt% Cu that were heat treated at 400 °C for 1 h and then water quenched. The particles in the matrix were identified as the Zn-precipitated phase. Submicron and nanoscale Zn particles were distributed in the Al matrix (Fig. 3). However, the Al–Zn alloy with Cu showed a different aspect ratio of the average particle size and distribution, i.e., the majority of Zn particles of the scale of a few 10 nm were uniformly distributed compared with the corresponding alloy without Cu. It is no wonder that the high Zn content in the Al matrix has a huge driving force for precipitation and and it could not help precipitation of Zn particles in the Al matrix despite its rapid cooling during water quenching after heat treatment because the supersaturated Al–Zn solution was thermodynamically unstable^20^. Therefore, the Cu addition to the Al–Zn alloy had a direct effect on the decrease in the average size of Zn precipitates during cooling after casting or solution heat treatment (Figs 2 and 3). Additionally, the Cu atoms were mostly observed in Zn particles instead of in the Al matrix (Fig. 3). Therefore, the Cu atoms were located as a solute in the Zn phase. The reason that Cu substitution in the Zn phase in the Al matrix caused decreasing particle size and uniform deformation must be related to the solution of Cu in the Zn phase. Despite the decreased particle size and uniform distribution, the ductility increase of the Al–33Zn–2Cu alloy was still not proven clearly (Fig. 1a). The grain boundary (GB) structures of the as-cast Al–35Zn and Al–33Zn–2Cu alloys are shown in Fig. 4. The heterogeneously nucleated particles were located at the GB (Fig. 4a and b); however, lamellar precipitates covered the GBs in the Al–33Zn–2Cu alloy. These lamellar precipitates were known to be discontinuous; therefore, the heterogeneously precipitated Zn particles that were mainly located at GBs were suppressed and alternated with discontinuous precipitation during solidification.

The ductility of the Al–33Zn alloy with Cu addition might be increased by the fast discontinuous precipitation that removed heterogeneous, large, precipitated particles affecting GB stability. Generally, the discontinuous precipitate was formed easily when a certain condition was satisfied; e.g., when there was a high driving force for precipitation, a large high-angle GB as a discontinuous precipitation site and an anisotropic crystal structure, which led to a different interfacial energy of the interface between precipitate and matrix^21–26^. The results of this study show that Cu addition reduces the interface energy between the Zn phase and Al matrix, which causes a drastic decrease in precipitate size and encourages discontinuous precipitation at GBs during solidification or solution treatment (see Supplementary Materials).

### Interface energy calculation by density functional theory

**Figure 5:**
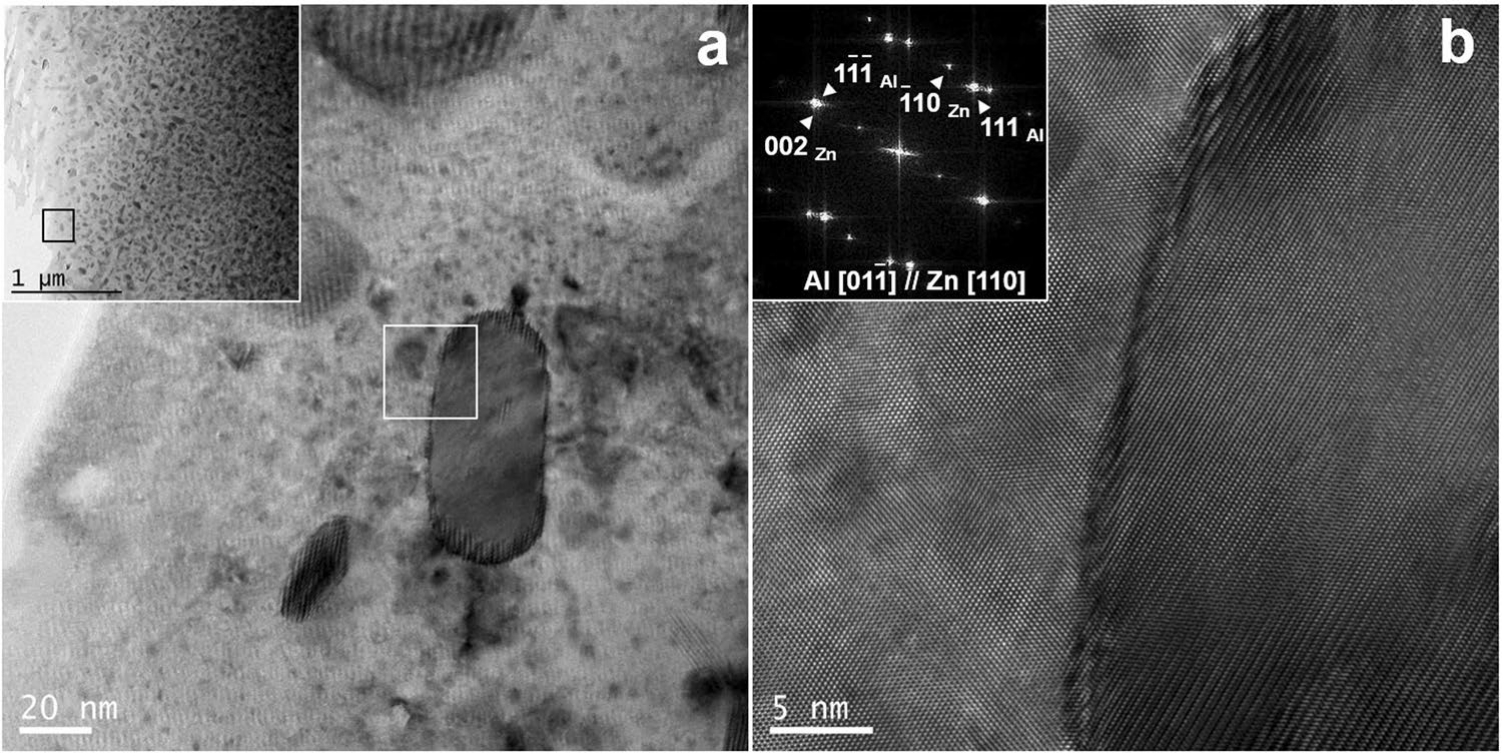
(**a**) TEM images of Zn precipitation in solution-treated Al–43Zn–2Cu alloy. The image in (**b**) represents the interface between the Al matrix and the Zn precipitate. The ellipsoidal Zn particle in the Al matrix has a coherent interface between Zn (0002) and Al (111); therefore, it was concluded that this coherent interface was the most occupied and stable interface among all other interfaces.

**Figure 6:**
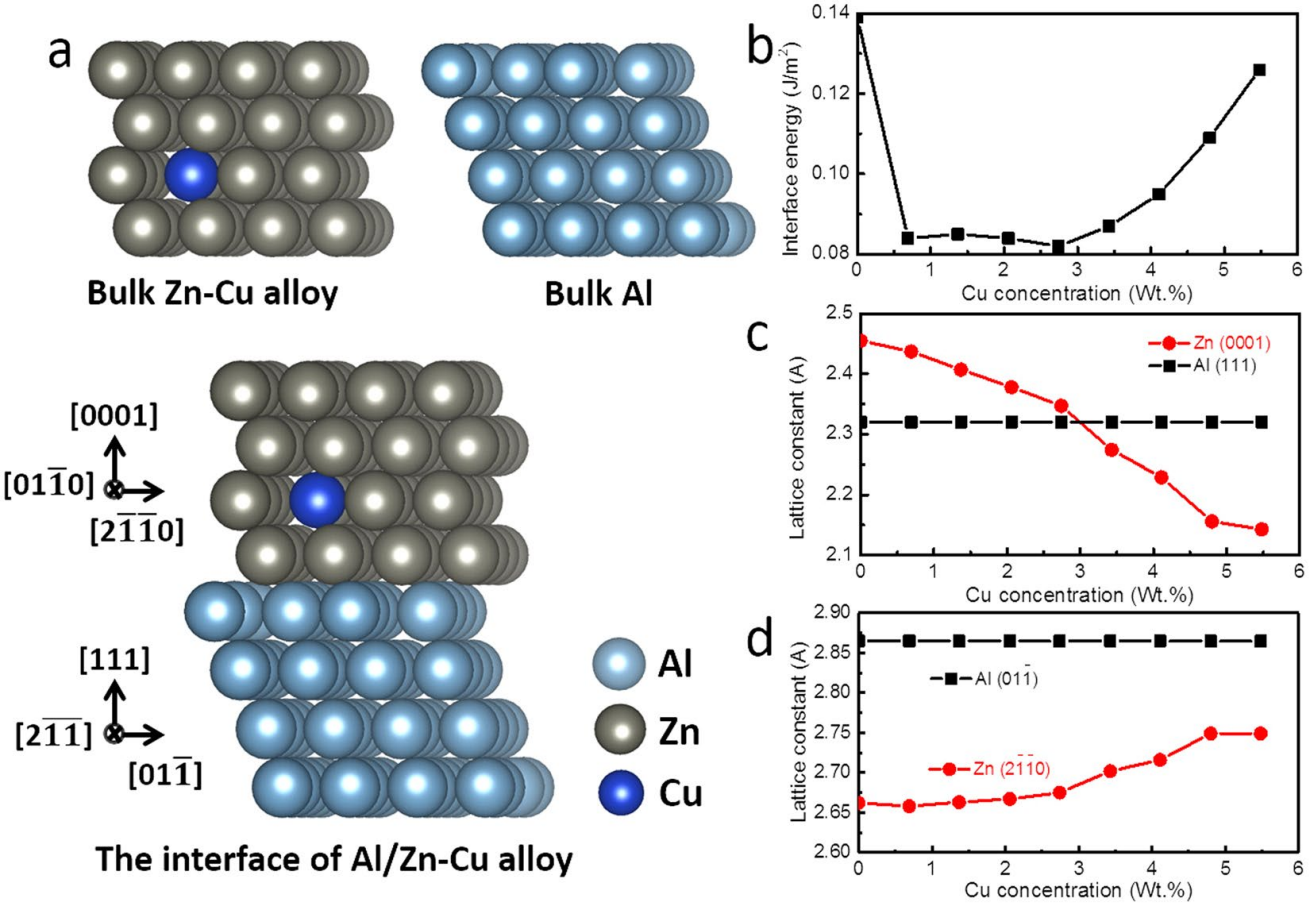
The stable geometries of (**a**) bulk Zn_44.3_Cu_0_._7_ alloy, bulk Al and the interface of Al/Zn_44_._3_Cu_0.7_; (**b**) the interface energy (E_inter_) (in J/m^2^) between Al and the Zn–Cu alloy as a function of the concentration of Cu ions. (**c**) The change in the lattice spacing of (0001)_Zn_ and (111)_Al_ planes, and (**d**) compared with that of ($2\bar{1}\bar{1}0$)_Zn_ and ($01\bar{1}$)_Al_ planes. The DFT calculation showed that the interface energy and lattice misfit between the Zn precipitate and the Al matrix decreased drastically by Cu substitution in Zn phases.

To elucidate why the Cu atoms in Zn precipitates change the interface energy in the Al–Zn alloy, first we verified the major interface usually generated between Zn and the Al matrix during precipitation. This interface had a coherent relationship with (111)_Al_//(0002)_Zn_ (Fig. 5); therefore, it is the most stable interface among all other crystallographic relationships. From Figs 2 and 3, the reason for the decrease in the size of Zn precipitates must be related to the decreasing interface energy between the interface of the Zn and Al matrix phase, particularly the substitution of Cu atoms in the Zn phase. Generally, the interface energy includes the chemical bonding and the strain energy at the interface. Weighing the interface energy by calculating the lattice misfit decrease from X-ray diffraction(XRD) or TEM results seems to be insufficient. To get a more accurate interface energy change, we performed first-principle calculations based on the DFT. The projector augmented-wave (PAW) method^27,28^ pseudopotentials were expanded with a cut-off energy of 417 eV, including 3d electrons as the valence states for Zn and Cu. We used the Perdew–Burke–Ernzerhof (PBE) approximation^29^ for the exchange-correlation potential as implanted in the Vienna ab initio simulation package code (VASP)^30,31^. For bulk and the (111)_Al_/(0001)_Zn_ interface system (Fig. 6a), k-points were generated by using the 7 × 7 × 7, 7 × 7 × 7 and 7 × 7 × 3 Monkhorst–Pack mesh, respectively.

Our calculations were performed to investigate the effect of Cu atoms on the interface between Al and Zn in the Al_55_Zn_45-x_Cu_x_ alloy. We assumed that all the incorporated Cu atoms substitute for Zn atoms. First, we compared the doping energies (*E*_*d*_) of a Cu atom in bulk Zn and the (111)_Al_/(0001)_Zn_ interface structure (Fig. 6a). The *E*_*d*_ can be calculated as ${E}_{d}={E}_{doping}-({E}_{pure}-{\mu }_{Zn}+{\mu }_{Cu})$, where *E*_*doping*_ and *E*_*pure*_ are the total energies of the system with and without a Cu atom, respectively, whereas *μ*_*zn*_ and *μ*_*cu*_ are the chemical potentials of Zn and Cu atoms obtained from bulk Zn and Cu, respectively. The doping energies of a Cu atom in bulk Zn and at the Al/Zn interface structure were found to be 0.21 and −0.56 eV, respectively, indicating that Cu atoms prefer to reside near the Al/Zn interface. Second, we obtained the interface energy (*E*_*inter*_) between (111)_Al_ and (0001)_Zn_ as the concentration of Cu increased up to 6 wt%. *E*_*inter*_ can be defined as shown in Eq. (1):

1 $${E}_{inter}=\frac{{E}_{Al/Zn(Cu)}-({E}_{Al}+{E}_{Zn(Cu)})}{A}$$

where *E*_*Al/Zn*(*Cu*)_, *E*_*Al*_ and *E*_*Zn*(*Cu*)_ are the total energies of the Al/Zn(Cu) interface structure, bulk Al and bulk Zn(Cu), respectively, and A is the area of the Al/Zn(Cu) interface. It is easier to form the interface when the *E*_*inter*_ is smaller. The *E*_*inter*_ of the Al/Zn(Cu) interface was reduced by about 0.06 J/m^2^ when the Cu concentration was between 1 to 3 wt% (Fig. 6b), whereas *E*_*inter*_ increases with a Cu concentration of 4 wt% or more. Thus, we predict that Cu atoms stabilize the Al/Zn interface and decrease the size of Zn particles in the Al matrix at the Cu concentration of 2 wt%, as shown in our experiments.

Using the DFT calculations, we extracted the lattice misfit of Al and the Zn_45−x_Cu_x_ alloy to examine the strain effect on the Al/Zn interface. The misfit between the lattice spacing of the (111)_Al_ and (0001)_Zn_ planes $({\rm{\delta }}=\{d{(111)}_{Al}-d{(0001)}_{Zn}\}/d{(111)}_{Al})$ was −6.0%, whereas that between the ($01\bar{1}$) Al and ($2\bar{1}\bar{1}0$) Zn planes was 7.1%, without the incorporation of Cu ions (Fig. 6c and d). When Cu ions are substituted for Zn, the distance between the (0001) Zn planes decreases and that for the ($2\bar{1}\bar{1}0$) Zn planes simultaneously increases. When the Cu concentration increased to 3 wt%, the lattice misfits for the (0001) and ($2\bar{1}\bar{1}0$) Zn planes became −0.2 and 6.3%, respectively. Thus, we expected that Cu ions play a role in reducing the strain applied to the Al/Zn interface up to the Cu concentration of 3 wt%.

## Discussion

The empirical and quantum mechanical calculation results showed that Cu addition increased both the strength and ductility of the cast or cast and subsequently solution-treated Al–Zn alloy. First, after adding Cu to the Al–Zn alloy, the Cu atoms dissolved preferentially in the precipitated Zn phase instead of in the Al matrix. Second, the Zn phase with dissolved Cu in the Al matrix decreased the interface energy between the Zn and Al phases, and led to a decreased critical particle size during cooling after casting or solutionizing compared with the corresponding alloy without Cu. Third, the low interface energy between the Zn and Al phases brought accelerated discontinuous precipitation at the GBs instead of the generation of large heterogeneous Zn precipitates, which was known as an important factor for decreasing ductility. Finally, the small size and uniform distribution of Zn particles in the Al matrix led to increasing strength by satisfying the particle-strengthening theory.

**Figure 7:**
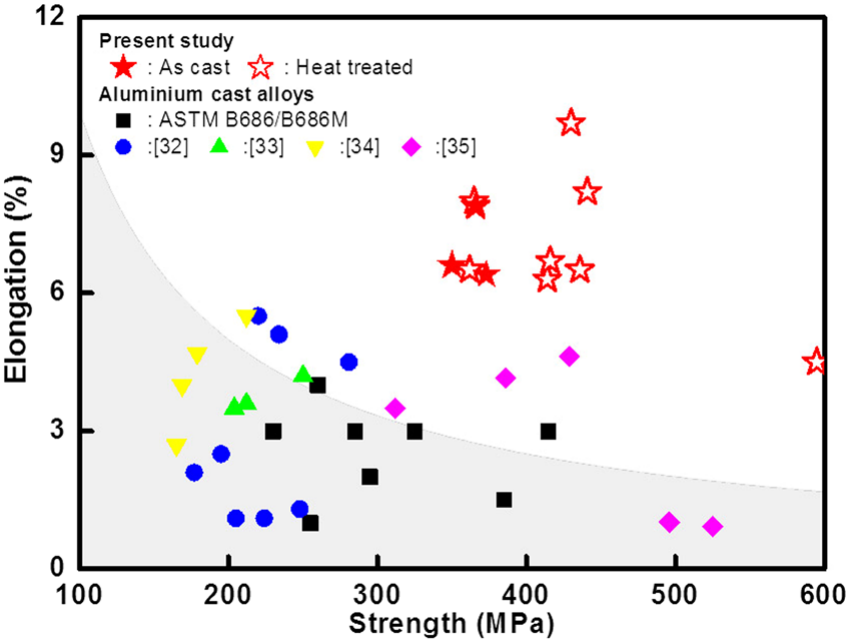
Comparison of the elongation and strength of our Al alloys with previously reported materials and a commercial Al alloy. Cu addition to the Al–Zn alloy shows an unusual combination of strength and elongation, which was obtained by decreasing precipitate size and GB stabilization.

Figure 7 shows the relationship between strength and ductility of conventional cast Al alloys and the alloy developed in this study^32–35^. We conclusively achieved our general objective of developing a high-strength and -ductile cast metal alloy by adding an appropriate element (in the case of this alloy, Cu), which leads to a decrease in interface energy. These mechanisms that control interface energy through the addition of a small amount of a second element, thereby improving strength and ductility, might be a key factor in developing many types of second-phase strengthening alloys, as well as the Al–Zn alloy.

## Methods

Fabrication of materials. Pure Al and Zn with 99.9% purity and Cu with 99.99% purity were used to prepare the materials investigated in this study. The nominal compositions are shown in Table 1. The Al–Zn alloy with and without additional Cu were fabricated to 25 mm-thick cast ingots using electric resistance furnace melting. To eliminate the segregation of the second element in the specimens, all Al–Zn alloys without or with Cu were subsequently solution heat-treated at 400 °C for 60 min. and then water quenched.

**Table 1:** Average composition of aluminum alloys used in this work.

Alloys	Al	Zn	Cu
Al-35Zn	Bal.	35.1	—
Al-33Zn-2Cu	Bal.	34.3	2.3
Al-45Zn	Bal.	43.4	—
Al-43Zn-2Cu	Bal.	42.4	2.55

Property evaluation and microstructural analysis. Tensile tests were performed with a gauge length of 10 mm at a nominal strain rate of 2 × 10^−1^/s on a universal testing machine (4206, Instron, USA). Grain morphologies and secondary-phase precipitates of the aged specimens were observed with a scanning electron microscope (SEM, JSM-6610LV; JEOL, Japan). The precipitates were investigated using a 200-kV field emission TEM (FE-TEM, JEOL-2100F; JEOL) equipped with an energy-dispersive X-ray spectroscopy detector (EDS), and a scanning TEM. TEM specimens were prepared to have a 3-mm diameter in the form of 60-μm-thick disk-type plates via mechanical polishing with a digitally enhanced precision specimen grinder (DEPS-101, Total Solution) and then ion-polished with a precision Ar ion polishing system (PIPS, model 691; Gatan, USA). The parallel and perpendicular sections for confirmation of the lamellar nature of discontinuous Zn precipitates were conducted with a dual-beam focused ion beam (FIB; Helios NanoLab, Netherlands).

## Electronic supplementary material

Supplementary Information
