# Supplementary material for: Simultaneous increase in strength and ductility by decreasing interface energy between Zn and Al phases in cast Al-Zn-Cu alloy
Source: Sci Rep. 2017 Sep 22;7:12195. doi: 10.1038/s41598-017-12286-7 (PMC5610273; doi:10.1038/s41598-017-12286-7)
Supplement: Supplementary file 1 — Supplementary Information [file 41598_2017_12286_MOESM1_ESM.pdf]

Supplementary materials for

Simultaneous increase in strength and ductility by decreasing interface energy between Zn and Al phases in cast Al-Zn-Cu alloy

Seung Zeon Han[1], Eun-Ae Choi [1], Hyun Woong Park[2], Sung Hwan Lim[2]*, Jehyun Lee[3], Jee Hyuk Ahn[1], Nong-Moon Hwang[4] and Kwangho Kim[5]*

[1]Structural Materials Division, Korea Institute of Materials Science, Changwon 642-831, Korea
[2]Department of Advanced Materials Science and Engineering, Kangwon National University, Chuncheon 200-701, Korea
[3]Department of Materials Science and Engineering, Changwon National University, Changwon 641-773, Korea
[4]Department of Materials Science and Engineering, Seoul National University, Seoul 151-744, Korea
[5]School of Materials Science and Engineering, Pusan National University, Busan 609-735, Korea

Seung Zeon Han and Eun-Ae Choi contributed equally to this work
*To whom correspondence should be addressed.
E-mail: shlim@kangwon.ac.kr and kwhokim@pusan.ac.kr

A. Supplement data of mechanical properties

Table S1. The tensile properties of as-cast and solution treated alloys shown in figure 1 and 7

Sample		Yield strength (MPa)	Tensile strength (MPa)	Elongation (%)
As cast	Al-35Zn	264.8	290.3	1.6
	Al-45Zn	267.7	286.1	3.5
	Al-33Zn-2Cu	279.3	366.3	7.2
	Al-43Zn-2Cu	309.7	350.5	2.7
After solution heat treatment	Al-35Zn	357.8	437.8	4.7
	Al-45Zn	293.0	358.2	9.1
	Al-33Zn-2Cu	423.2	518.8	7.6
	Al-43Zn-2Cu	519.3	599.8	3.4

B. Supplement data of precipitation density

Table S2. The density of precipitations in Al-Zn and Al-Zn-Cu alloys shown in Fig.2.

**Sample**	**Al-35Zn**	**Al-33Zn-2Cu**	**Al-45Zn**	**Al-43Zn-2Cu**
**Density of precipitation ($\times 10^{10}/cm^2$)**	**3.85**	**5.58**	**2.26**	**6.15**

C. Observation of major interface relationship

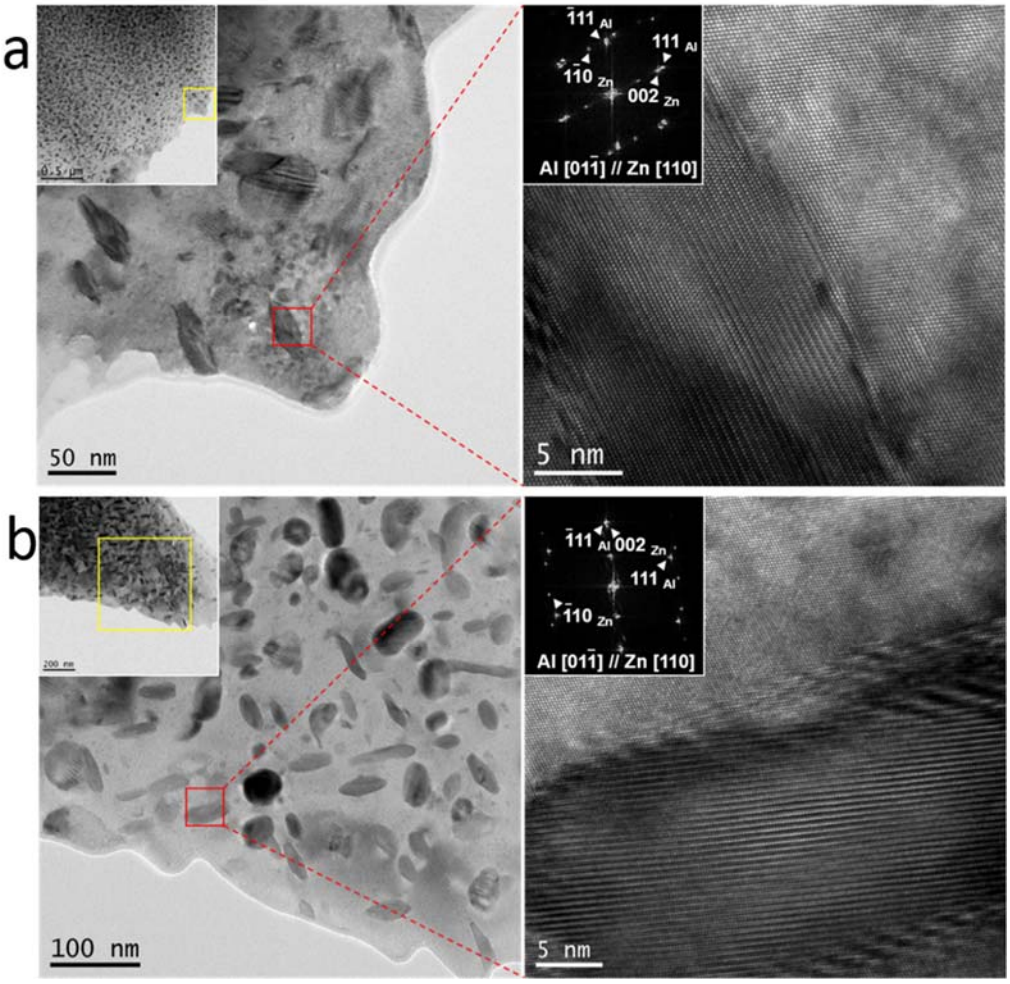

Figure S1. The coherent interface relationship between $(111)_{Al}$ and $(0002)_{Zn}$, as shown in figure 5, was easily observed at any region (a) and other specimen (b), therefore it could be concluded that this interface had larger area fraction compared to the interfaces with other crystallographic relationship.

D. Observation of fracture surface after tensile test

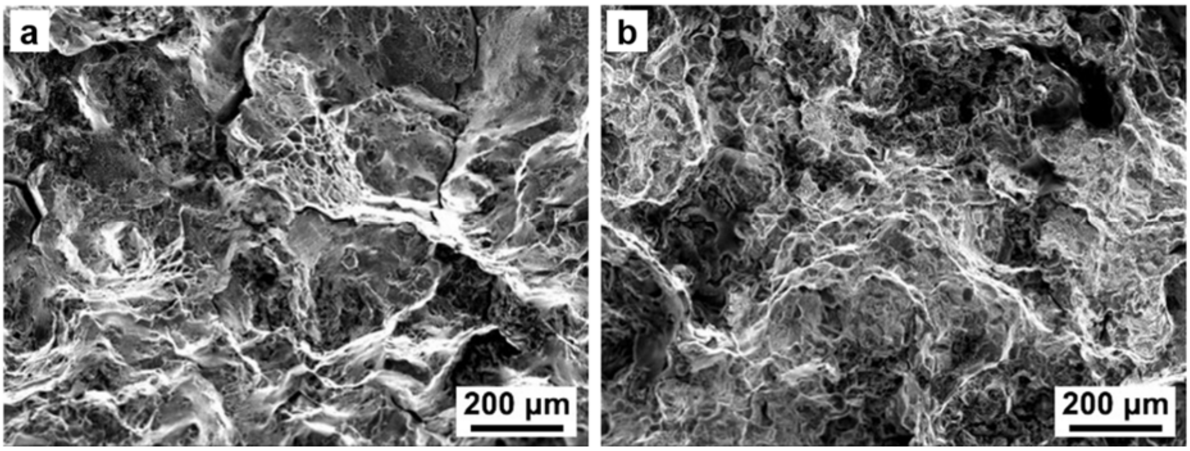

Figure S2. The fracture surfaces of as-cast (a) Al-35Zn and (b) Al-33Zn-2Cu alloys showed that the addition of 2% Cu tended to encourage ductile fracture, as associated with the increased number of precipitates. It was therefore confirmed that the addition of Cu into Al-35Zn alloy increased ductility along with the enhanced tendency for dimpled rupture mode.

E. The results of DFT calculations for Cu ions with various distribution patterns.

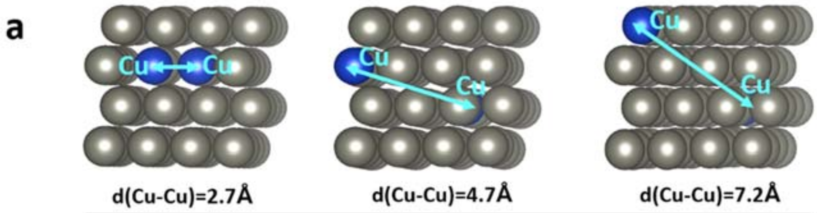

Sample	1	2	3
d(Cu-Cu) (Å)	2.7	4.7	7.2
E_{form} (meV)	73.1	38.2	-75.2
d(0001) (Å)	2.40	2.41	2.40
d($2\overline{1}\overline{1}0$) (Å)	2.66	2.66	2.66

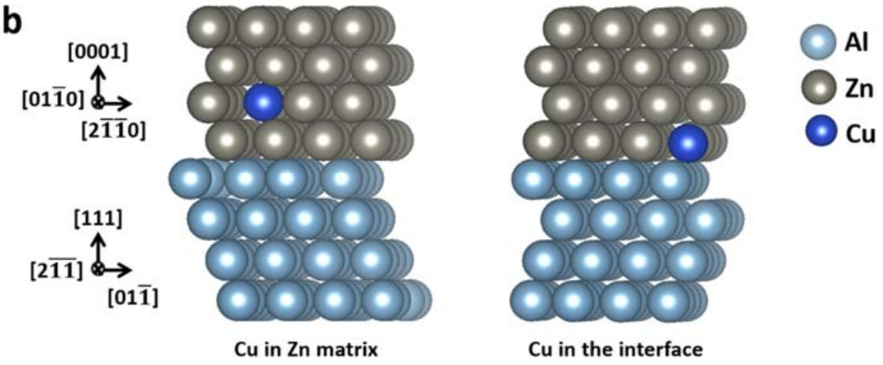

Sample	1	2
Cu location	in Zn matrix	in the interface
E_{inter} (mJ/m^2)	84.4	84.3

Figure S3. (a) Formation energies and lattice constants according to the distance of two Cu in Zn matrix. Here, two Cu atoms were substituted for Zn in Zn 64-atom cell. The concentration of Cu is 1.37 wt.%. The formation energy (E_{form}) is calculated as $E_{form} = E_{Total} - (62\mu_{Zn} + 2\mu_{Cu})$, where E_{Total}, μ_{Zn} and μ_{Cu} are the total energy of the system and the chemical potentials of Zn and Cu atoms obtained from bulk Zn and Cu, respectively. As the distance between the two Cu ions increases to 7.2Å, the E_{form} decreases to -75.2meV, indicating that Cu in Zn matrix forms a substitutional solid solution. Thus, we position the Cu atoms in Zn matrix so that the distance between them is as far as possible in all calculations. In the other hand, the position of Cu has little effect on the lattice constants. (b) The interface energies (E_{inter}) according to the position of a Cu ion in $(111)_{Al}/(0001)_{Zn}$ interface structure. The E_{inter} is almost unchanged whether Cu is in Zn matrix or in the interface.
